# Editorial: The NeuroCOVID-19 syndrome: cognitive and psychological profiles, physiopathology, and impact on neurologically vulnerable populations

**DOI:** 10.3389/fneur.2024.1452895

**Published:** 2024-07-19

**Authors:** Caroline Jose, Thorsten Rudroff, Ludivine Chamard-Witkowski

**Affiliations:** ^1^Centre de Médecine de Précision, Centre Hospitalier Universitaire Georges-L.-Dumont, Réseau de Santé Vitalité, Moncton, NB, Canada; ^2^Département de Médecine de Famille et de Médecine d'urgence, Faculté des Sciences de la Santé, Université de Sherbrooke, Sherbrooke, QC, Canada; ^3^Département de Chimie et de Biochimie, Faculté des Sciences, Université de Moncton, Moncton, NB, Canada; ^4^Department of Neurology, University of Iowa, Iowa City, IA, United States; ^5^Centre de formation médicale du Nouveau-Brunswick, Moncton, NB, Canada

**Keywords:** long COVID, sequelea, cognitive dysfunction, neurological involvement, NeuroCOVID, imaging, biomarkers, physiopathological mechanism

## 1 Introduction—From COVID-19 to NeuroCOVID

The Coronavirus Disease 2019 (COVID-19) global pandemic, caused by Severe Acute Respiratory Syndrome Coronavirus 2 (SARS-CoV-2), has led to the identification of a broad range of post-acute COVID-19 neurological symptoms including cognitive impairments, executive dysfunctions, changes in sleep, emotional distress, pain and fatigue (1). Alarmingly, those post-acute sequelae of COVID-19 (PASC) can occur several weeks after infection, arise after severe, mild, or even asymptomatic SARS-CoV-2 infection, and are characterized by the persistence, worsening, or new onset of chronic and debilitating neurological symptoms, which have led to the use of NeuroCOVID syndrome terminology (2, 3).

Despite a global research effort in describing PASC, there are still many research challenges and many open questions, particularly relating to pathophysiology, specific biomarkers, effective treatments (3) and risk factors of cognitive deficits and other neurological manifestations (4).

The goal of this Research Topic was to consolidate and deepen our actual knowledge on cognitive and neuropsychological symptoms in NeuroCOVID syndrome. We highlight below the unique contributions to the Research Topic of 10 articles (seven reviews, one original article, and one case report), and discuss their findings in the broader context of the methodological pitfalls, knowledge gaps and future research needs in post-acute sequelae of COVID-19 (referred interchangeably to post-acute COVID-19 syndrome, PASC or NeuroCOVID in this editorial article).

## 2 Toward a comprehensive pathological understanding

Li et al. provide a broad perspective on the clinical and basic evidence of cognitive impairment following COVID-19 through an overview of the latest neuropsychological and neuroimaging findings. They then detail the five mechanisms by which COVID-19 may impair cognitive function and lead to memory loss, namely through (1) direct viral damage to the central nervous system (CNS), and indirect mechanisms such as (2) inflammation effects, (3) vascular and hypoxic changes, (4) metabolic impacts, and (5) immune response.

Interestingly, Bouhamdani et al. describe a unique case presentation of *Nocardia farcinica* cerebral abscess in a male patient with sudden immunodeficiency several months after mild COVID-19. This case strengthens the notion of immunomodulation after COVID-19 and well after the viral infection has cleared, and draws attention to the need for timely consideration of opportunistic infections for patients with a history of COVID-19.

Focusing on inflammation effects, Saucier et al. propose that disturbance of reactive microglia and astroglia potentially contribute to neurological impairment after COVID-19. They further illustrate an indirect pathway, where microglial activation and neuroinflammation are consequential repercussions of systemic inflammation induced by the SARS-CoV-2 virus infection and resulting in the blood-brain-barrier breakdown. They propose that the translocation of peripheral cytokines and immune cells to the CNS culminates in microglial activation and brain neurological damage.

Strikingly, in their original research article, Havdal et al. found no evidence of ongoing neuroinflammation, and neither brain injury biomarkers nor neurocognitive test results that were associated with subjective reported symptomatology. The discrepancy between subjective symptoms and objective findings adds to a growing body of evidence suggesting that PASC may be associated with functional CNS alterations and have origins more related to a combination of biological, psychological and social factors, rather than being solely biomedical in nature.

Even more surprising, they report that the SARS-CoV-2 infection status was not associated with PASC. Indeed, the percentage of PASC in the non-infected and in the infected groups were almost equal, at 47 and 48%, respectively, 6-month after the infection or baseline assessment. While some study limitations might partly explain these findings, Lavin et al. also discuss the possibility that the symptoms of PASC, notably pain, may arise independently of neuronal damage and/or interoceptive afferent signals, referring to published evidence of psychosocial factors as important predictors of persistent symptoms in PASC, at least in a subset of the patient population.

Therefore, it is still to be established whether the subjective experience of neurological and neuropsychological symptoms in PASC correspond with objectively measurable deficits.

## 3 The search for biomarkers

To determine the incidence of the common neurological abnormalities using magnetic resonance imaging (MRI) in patients with severe COVID-19, Boparai et al. conducted a meta-analysis including 32 studies. They report the incidence of any MRI abnormality to be 55%, with most injuries appearing to be of vascular origin. They note, however, that the presentation of brain injury was diverse among the studies with no substantial pattern of injury emerging. Moreover, their analysis of the association between MRI abnormalities and clinical findings further confirms that there are likely many mechanisms, both direct and indirect, by which brain injury occurs in COVID-19 patients.

Both Okrzeja et al. and Cull et al. reviewed findings obtained with several neuro-imaging modalities to identify NeuroCOVID-specific biomarkers. Okrzeja et al. reviewed neuroanatomical findings from three imaging modalities and their utility in differential diagnoses, namely, magnetic resonance imaging (MRI), positron emission tomography/computed tomography (PET/CT; and more specifically 18F-FDG-PET/CT), and computed tomography (CT). Notably, they highlighted the potential pathophysiological link between PASC and Guillain-Barré syndrome based on MRI findings that needs to be further explored. Cull et al. expanded their review to describe findings obtained with other advanced imaging techniques, such as SPECT imaging, 18F-Amyloid-PET/CT, structural MRI, functional MRI, diffusion MRI, and Susceptibility-weighted imaging.

Both studies highlight the potential of hypometabolism, as revealed by 18F-FDG-PET/CT in a subset of studies, as a quantitative marker of cerebral damage of post-COVID-19 syndrome. However, limitations and inconsistent findings highlighted by those two reviews led both teams to suggest combining different neuro-imaging modalities, from structural imaging to functional and metabolic imaging, to identify more specific and sensitive neurological markers of NeuroCOVID.

Overall, although numerous radiological findings have been reported in PASC patients, few studies have been able to link the brain lesions to the PACS symptoms. Comeau et al. reached similar conclusions from their review of studies using blood, plasma and/or cerebrospinal fluid biomarkers of neuronal injury and of inflammatory processes in PASC patients, stating that usefulness of the liquid biomarkers studied so far remains tenuous because of the heterogeneity of findings and of our insufficient state of knowledge on PASC.

## 4 The blurred line with neurodegenerative diseases

Complicating the understanding of NeuroCOVID pathophysiology is the symptoms' overlap with other neurological conditions (4).

The review published by Shajahan et al. explores the complex interrelationships between COVID-19 and Alzheimer's disease. They explain that Alzheimer's pathological terrain, such as increased proinflammatory cytokines, NLRP3 activation, and oxidative stress, even during its asymptomatic phase, could be a risk factor for severe neurological impact of SARS-CoV-2 infection. On the other hand, COVID-19 could trigger the onset of Alzheimer's disease by modulating pathological pathways in the brain that are common between both diseases through the direct or indirect mechanisms described by Li et al.

## 5 Summary and future directions

Through this Research Topic, we hoped to improve the knowledge of the risk factors and physiopathology of the NeuroCOVID cognitive deficits and other neurological manifestations. While there is still much more research that needs to be done to reach that goal, we believe that this Research Topic will initiate discussions on the psychosocial involvement as well as on the predictive value of objective neurological biomarkers in NeuroCOVID research and care.

From this Research Topic, we can draw three main conclusions:

- A complete and clear picture of the NeuroCOVID syndrome is hampered by the non-specific nature of the majority of clinical manifestations in the PASC spectrum, the lack of relevant control groups in most studies, and other methodological issues, such as small sample sizes or heterogeneous samples.
- The development and validation of biomarkers that can be employed for the prediction, diagnosis and prognosis of NeuroCOVID will stem from studies combining multimodal neuroimaging, liquid biomarkers investigation, and a thorough clinical assessment (including medical history, comorbidities and neuropsychological testing).
- The state of knowledge on NeuroCOVID lags behind the increasing care needs for patients experiencing PASC because of the varied factors impacting the clinical trajectories of both acute and long COVID-19 (clinical, Sociodemographic, genetic, psychosocial, environmental, ect.).

Large scale and multidisciplinary studies adequately designed to stratify the PASC population in subgroups of PASC symptoms profiles could help to better refine the risk factors associated with each of those different COVID-19 trajectories. Those subgrouping efforts could eventually guide the development of precision medicine and precision public health for the management of this post-COVID-19 clinical reality and of future pandemics.

## Author contributions

CJ: Conceptualization, Writing – original draft, Writing – review & editing. TR: Writing – review & editing. LC-W: Writing – review & editing.
